# The impact of rifampin on the efficacy of implant retention and prosthesis removal in staphylococcal periprosthetic joint infection

**DOI:** 10.3389/fcimb.2025.1587436

**Published:** 2025-07-16

**Authors:** Canhong Zhang, Juncheng Li, Lan Lin, Mingzhong Liu, Yang Chen, Yuanqing Cai, Jiexin Huang, Zida Huang, Chaofan Zhang, Wenming Zhang, Xinyu Fang, Wenbo Li

**Affiliations:** ^1^ Department of Orthopedic Surgery, The First Affiliated Hospital of Fujian Medical University, Fuzhou, China; ^2^ Department of Orthopaedic Surgery, National Regional Medical Center, Fujian Medical University, Fuzhou, China; ^3^ Fujian Provincial Institute of Orthopaedics, the First Affiliated Hospital, Fujian Medical University, Fuzhou, China; ^4^ Department of Orthopedic Surgery, Quanzhou First Hospital Affiliated to Fujian Medical University, Quanzhou, China; ^5^ Department of Orthopaedic Surgery, Nanping First Hospital Affiliated to Fujian Medical University, Nanping, China

**Keywords:** periprosthetic joint infection, staphylococci, rifampin, implant retention, prosthesis removal

## Abstract

**Objectives:**

The purpose of this study was to evaluate the impact of adjunctive rifampicin therapy on the outcomes of prosthesis retention versus removal in patients with staphylococcal prosthetic joint infection (PJI) undergoing antibacterial treatment.

**Methods:**

A retrospective study was conducted on 227 patients diagnosed with Staphylococcal PJI from March 2014 to September 2023 who underwent debridement, antibiotics, and implant retention (DAIR) or explantation and revision surgery. Based on antimicrobial susceptibility testing, we used an effective baseline antibiotic regimen. We defined the combination of this regimen with rifampicin as the “rifampicin treatment group” and the regimen without rifampicin as the “non-rifampicin treatment group”.

**Results:**

A total of 79 patients were included in the rifampin treatment group and 148 in the non-rifampin treatment group. There was no significant difference in the remission rate of PJI between the rifampin treatment group and the non-rifampin treatment group (79.75% vs 73.65%, p = 0.083). Additionally, Kaplan-Meier survival curve analysis showed no statistically significant difference between the two groups (p = 0.509). However, the incidence of drug-related adverse events was significantly higher in the rifampin treatment group compared to the non-rifampin treatment group (31.65% vs 8.78%, p < 0.001). There were no significant difference in treatment success rates between the use and non-use of rifampin in DAIR, one-stage revision, or two-stage revision, as well as in hip or knee joints. Binary logistic regression analysis identified diabetes and active smoking as independent significant risk factors for treatment failure, while rifampin was not an independent risk factor affecting the outcome.

**Conclusion:**

The study has not demonstrated that the standard antibiotic regimen combined with rifampin has a significant effect on the efficacy of retaining or removing prostheses in staphylococcal PJI, but rather increases drug-related adverse events. Standard surgical procedures, accurate pathogen diagnosis, and treatment are particularly crucial in the management of PJI.

## 1 Introduction

Arthroplasty is an effective treatment for end-stage joint diseases. It is predicted that by 2030, the United States will perform 572,000 and 3.48 million hip and knee arthroplasties, respectively (Helou et al., 2010). Among the many complications of arthroplasties, prosthetic joint infection (PJI) is the most catastrophic (Yoon et al., 2017). The incidence of PJI is 1%-2% in primary hip arthroplasties and 2%-3% in primary knee arthroplasties (Aggarwal et al., 2014; Renz and Trampuz, 2015). PJI imposes a significant burden on patients and incurs substantial costs to the healthcare system (Tande and Patel, 2014).

The Musculoskeletal Infection Society (MSIS) criteria for the diagnosis of PJI were established in 2011 to define PJI (Parvizi et al., 2011). For acute infections, the debridement, antibiotics, and implant retention (DAIR) procedure is frequently utilized. However, for chronic infections or as a salvage procedure, prosthesis removal and revision surgery are commonly performed, either as one-stage or two-stage revision surgery (Anemüller et al., 2019). *Staphylococcus aureus* and coagulase-negative staphylococci (CoNS) are the most common causative agents of PJI, accounting for 30-47% and 12-44% of cases, respectively (Osmon et al., 2013; Lora-Tamayo et al., 2013; Widmer et al., 1990; Zimmerli et al., 1998). A significant challenge in treating PJI is the presence of biofilms on the implant surface, which protect bacteria from host immune responses and antimicrobial agents. Another challenge is the invasion of bacteria into osteoblasts and osteocytes, leading to intracellular infection, which can cause persistent or recurrent infections. Staphylococcus species are prone to forming biofilms (Aydın et al., 2021), and *Staphylococcus aureus* can also survive, reproduce, and persist intracellularly (Schröder et al., 2006).

Rifampicin is a semi-synthetic antibiotic that specifically inhibits bacterial RNA synthesis, with the β-subunit of RNA polymerase being the primary target of rifampicin (Wehrli, 1983). Due to its biofilm activity, rifampicin is considered one of the most important antibiotics for treating PJI (Cobo and Del Pozo, 2011). Rifampicin not only possesses anti-biofilm properties but can also accumulate intracellularly and eliminate most intracellular bacteria (Mohamed et al., 2014). Zimmerli et al. conducted a randomized controlled trial, concluding that a fluoroquinolone combined with rifampicin was the most effective antibiotic combination therapy for staphylococcal PJI (Widmer et al., 1990). Since then, the use of rifampicin in the treatment of staphylococcal PJI has become increasingly popular, especially in many European hospitals (Zimmerli et al., 1998). Beldman et al.’s multicenter study found that antibiotic therapy including rifampin had significant advantages, with the treatment failure rate increasing from 32.2% with rifampin to 54.2% without it (Beldman et al., 2021).

Although many studies have reported benefits associated with rifampin, its necessity remains a subject of debate (Aydın et al., 2021). A multicenter randomized controlled trial conducted in Norway in 2020 questioned the efficacy of rifampin for DAIR treatment of acute Staphylococcal PJI, and this study could not clearly demonstrate the advantage of adding rifampin (Karlsen et al., 2020). For one-stage and two-stage revisions, since the prosthesis has been entirely removed, there is still much debate about whether rifampicin can effectively reduce the failure rate of treatment. In a multicenter observational study by Kramer et al., which included 375 cases of staphylococcal PJI and underwent one-stage and two-stage revisions, a statistically significant benefit of rifampicin was only observed in chronic cases treated with two-stage revision, but no benefit was observed in one-stage revision and total cohort (Kramer et al., 2023). Rifampicin, as a strong inducer of cytochrome enzymes in the liver and intestines, also induces transport proteins in the gut. Therefore, it has a multitude of drug interactions (Baciewicz et al., 2013). For patients with cardiovascular or metabolic issues, drug interactions have the potential to destabilize hemodynamic indicators, which can lead to severe complications such as hypotension and arrhythmias.Thus, whether rifampin should be used in combination during the treatment of PJI warrants further investigation.

With this in mind, we conducted a retrospective study of patients diagnosed with staphylococcal PJI from March 2014 to September 2023, who underwent DAIR or prosthesis removal revision surgeries (one-stage and two-stage revisions), to observe the impact of including rifampicin in sensitive antibiotic regimens on the success rate of treating staphylococcal PJI.

## 2 Materials and methods

### 2.1 Patient selection and clinical data collection

This study included patients with staphylococcus PJI treated at our center from March 2014 to September 2023. The diagnosis of PJI was made using the 2011 criteria from the MSIS (Parvizi et al., 2011). The diagnosis was jointly determined by orthopedic surgeons, microbiologists, and infectious disease specialists based on this criteria.

**Figure 1:**
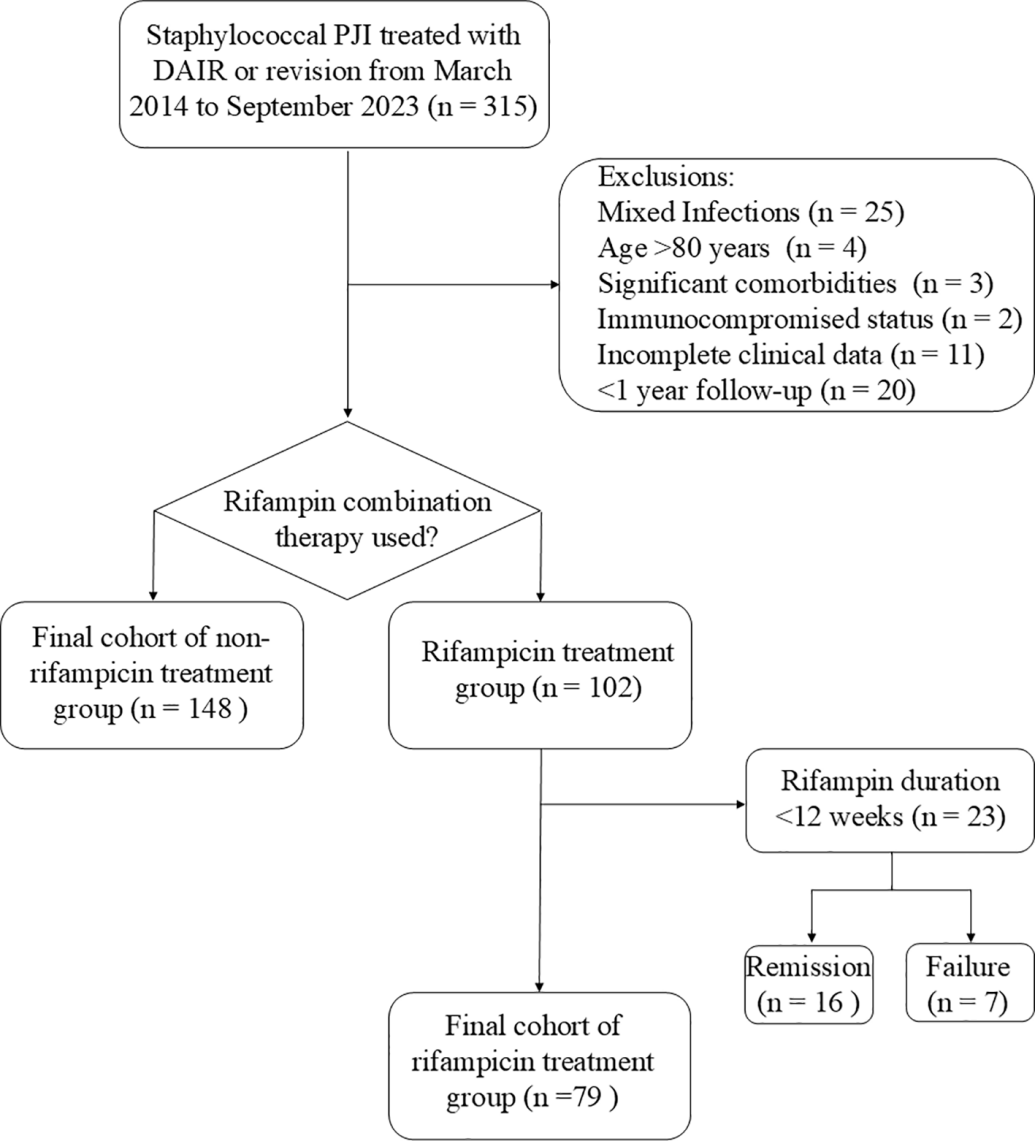
Flow diagram of patients enrollment.

The inclusion criteria were: 1) diagnosed with PJI caused by staphylococcus, and treated with DAIR, one-stage revision, or two-stage revision; 2) with comprehensive clinical data available; 3) patients were under 80 years of age, without severe comorbidities or a state of immunosuppression; 4) the patients demonstrated strong adherence to the treatment plan and were committed to attending regular follow-up appointments. Exclusion criteria were: 1) patients had other bacterial infections or mixed infections; 2) patients with PJI did not undergo DAIR, one-stage or two-stage revision; 3) patients with poor compliance who had difficulty attending regular follow-up visits; 4) resistant to rifampin, with inadequate rifampin treatment, including monotherapy, insufficient dosage, and a treatment duration of less than 12 weeks (**Figure 1**).

We extracted data on the patients’ demographic information, laboratory results, microbiological culture results, treatment regimen, medication regimen, complications, infection control, and antibiotic-related adverse events. This retrospective study was approved by our hospital ethics committee.

### 2.2 Surgical technique

#### 2.2.1 DAIR

Once a patient was diagnosed with acute PJI, we administered empirical antibiotics after joint aspiration and proceeded with DAIR as soon as possible. The principal steps of the surgery were as follows: 1) in all DAIR procedures, we removed the polyethylene liner or femoral head; 2) a thorough debridement was performed to excise all infected and necrotic tissue, after which the wound was soaked with povidone-iodine for 30 minutes and then irrigated with 6–9 liters of saline using a pulsatile lavage device; 3) the wound was temporarily closed, the surgical field was re-disinfected, draped, and new instruments were used for subsequent operations; a new liner or femoral head was implanted. Drains were typically left in place for 2 days.

#### 2.2.2 One-stage revision

The primary surgical procedures were as follows: 1) complete removal of bone cement and infected prostheses was undertaken; 2) a thorough debridement of infected soft tissues and bone was performed, followed by irrigation with 6–9 liters of saline solution; 3) prior to the implantation of new prostheses, it was imperative to change to a new set of surgical instruments. Patients who underwent cemented (Heraeus, Germany) implantation had their cement mixed with vancomycin and sensitive antibiotics (Wu et al., 2024).

#### 2.2.3 Two-stage revision

During the first-stage surgery, the steps for prosthesis removal and debridement were similar to those in a one-stage revision. While a new prosthesis is typically implanted in a one-stage revision, in this case, a spacer was implanted instead. The bone cement within the spacer was applied during the doughy phase to prevent excessive bone loss during the subsequent surgery, and was mixed with vancomycin and sensitive antibiotics.

After a minimum of 4 weeks of antibiotic holiday, a joint assessment was conducted by infectious disease specialists and orthopedic surgeons. Patients who exhibited no signs of infection and had negative erythrocyte sedimentation rate (ESR) and C-reactive protein (CRP) were considered to have had their infection eradicated, and were thus candidates for the second-stage surgery involving prosthesis reimplantation. Intraoperatively, all components and bone cement were removed, and periprosthetic tissues were sent for frozen section analysis. If the infection was not eradicated, debridement was performed followed by the implantation of an antibiotic-laden bone cement spacer. Otherwise, a new prosthesis was implanted.

### 2.3 Microbial culture

Standard incubation for microbial cultures was conducted for 7 days; however, in instances where results were negative or where there was suspicion of low-virulence pathogens, the incubation period was extended to 14 days. To optimize the yield of positive microbial cultures, a standardized protocol for cultivation was meticulously adhered to, as detailed subsequently (Hu et al., 2024).

Joint aspiration was performed on patients suspected of having PJI prior to surgery. The collected synovial fluid was rapidly injected into Bactec Plus/F aerobic and anaerobic blood culture bottles (BD, Germany) and subsequently placed into a Bactec 9050 incubator (BD, Germany) for culture.

During tissue collection, a blade was utilized for incision to avoid the use of electrocautery, and 5 periprosthetic tissue biopsies were conducted throughout the surgery. The tissue specimens were placed in sterile EP grinding tubes with 1 ml of brain heart infusion broth (Qingdao Haibo Biotechnology Co., Ltd., China) and then homogenized in an automatic rapid grinder (JXFSTPRP-24, Shanghai Jingxin Industrial Development Co., Ltd., China) at 40 Hz for 60–90 seconds. Subsequently, the homogenate was inoculated onto blood agar plates (Thermo Fisher Scientific, USA) and cultured under both aerobic and anaerobic conditions.

The explanted prosthesis was placed in a sterile container filled with saline solution, and then subjected to ultrasonication (40 kHz, 5 minutes) to disrupt the biofilm on the surface of the prosthesis. Following this, the sample was centrifuged for 5 minutes at 4,000 revolutions per minute (rpm). The supernatant was discarded, and the pellet was resuspended in sterile saline solution before being injected into blood culture bottles for incubation.

For cases with challenging cultures, to further enhance the accuracy of culturing and to rule out the possibility of polymicrobial infections, metagenomic next-generation sequencing (mNGS) was employed as an adjunct diagnostic tool for such patients. Intraoperatively obtained synovial fluid, tissue, or sonicated fluid samples were confirmed through various methods to corroborate mNGS results, including optimized microbial culture techniques and polymerase chain reaction (PCR) detection.

### 2.4 Antibiotic regimen

For patients undergoing DAIR procedures, an empirical antibiotic treatment regimen was initiated with vancomycin (1.0 g ivgtt q12 h) in combination with meropenem (2.0 g ivgtt q8 h) until culture and mNGS results were obtained. Subsequently, the regimen was tailored to pathogen-specific antibiotics based on susceptibility results. Intravenous antibiotics were administered for 2 weeks, followed by a transition to oral antibiotics for an additional 10 weeks.

For both one-stage and two-stage revisions, we tailored the antibiotic regimen based on culture and susceptibility results. Patients with methicillin-susceptible bacteria were treated with intravenous cefazolin, while those with methicillin-resistant bacteria received intravenous vancomycin. Following 2 weeks of intravenous antibiotics, patients were switched to oral antibiotics for an additional 10 weeks.

The criteria for using baseline antibiotic alone were as follows: 1) Bacterial culture and susceptibility testing demonstrated sensitivity to baseline antibiotic; 2) Patients were unwilling to receive rifampin therapy or had poor adherence to rifampin; 3) Some patients had documented allergies to rifampin or had comorbidities that precluded the use of rifampin therapy; 4) As this was a retrospective study, some patients had been treated with a single antibiotic prior to the incorporation of rifampin into our center’s standard treatment protocol for PJI. The criteria for the addition of rifampin to the regimen are as follows: 1) Bacterial culture and susceptibility testing demonstrated sensitivity to both baseline antibiotic and rifampin; 2) Patients were willing to receive rifampin therapy and had good adherence to the treatment regimen; 3) The patient’s overall health status was suitable for rifampin treatment.

In the rifampin treatment group, we typically added rifampin to the oral antibiotic regimen 2 weeks post-surgery when switching from intravenous to oral antibiotics. If the patients tolerated it, a dosage of 600mg once daily was administered, with the duration of rifampin therapy lasting for 12 weeks. Total antibiotic duration was 14 weeks.

### 2.5 Outcome evaluation

Treatment success was defined as the relief of clinical symptoms, no recurrence of infection, no new infections, and no long-term antibiotic suppression during the 12-month follow-up period. Treatment failure was defined as the need for further surgery due to infection (such as a second surgical debridement, implant removal, or amputation), PJI-related mortality, or the requirement for long-term antimicrobial therapy due to persistent infection-related clinical symptoms.

Antibiotic-related adverse events were classified as follows: 1) myelosuppression, defined as a leucocyte count > 4 × 10^9^/L before the use of antibiotics and a leucocyte maximum < 3 × 10^9^/L during antibiotic treatment; 2) hepatotoxicity, indicated by an aspartate aminotransferase (AST) or alanine aminotransferase (ALT) peak greater than 1.5 times the baseline normal pre-treatment value; 3) nephrotoxicity, defined as a serum creatinine level exceeding 1.5 times the baseline normal pre-treatment level; 4) gastrointestinal symptoms (Xu et al., 2022).

### 2.6 Statistical analysis

SPSS 27.0 (IBM, New York, USA) was utilized for data analysis. GraphPad Prism 10 (GraphPad Software, San Diego, USA) was employed for the creation of graphs. Continuous variables were calculated as mean and standard deviation (SD), and analyzed with independent-samples t-test if they followed a normal distribution. If they did not follow a normal distribution, the Mann-Whitney U test was used and expressed as medians (interquartile ranges (IQRs)). Categorical variables were assessed using Pearson’s chi-square tests, with adjustments for continuity or Fisher’s exact probability tests when necessary. Logistic regression analysis was conducted to identify the independent risk factors associated with adverse outcomes. Kaplan-Meier survival analysis and the log-rank method were used to assess infection control. A p-value < 0.05 was considered statistically significant.

## 3 Results

### 3.1 Demographic characteristics

**Table 1** presents the general demographic characteristics of the rifampin treatment and non-rifampin treatment groups in this study. In the rifampin treatment group, there were 79 patients with an average age of 65.44 years (standard deviation (SD) 9.82), comprising 41 males and 38 females. Among these patients, 30 cases were infected with *Staphylococcus aureus*, of which 3 were methicillin-resistant *Staphylococcus aureus* (MRSA) and the remaining 27 were methicillin-susceptible *Staphylococcus aureus* (MSSA). The other 49 cases were infected with Coagulase-negative staphylococci (CoNS), with 7 being methicillin-resistant Coagulase-negative staphylococci (MR-CoNS) and the rest 42 being methicillin-susceptible Coagulase-negative staphylococci (MS-CoNS). In terms of surgical procedures, DAIR, one-stage revision and two-stage revision were performed in 33 cases, 21 cases, and 25 cases, respectively. The non-rifampin treatment group consisted of 148 patients, with an average age of 65.12 years (SD 10.43), including 66 males and 82 females. Among them, 60 cases were infected with *Staphylococcus aureus*, with 5 being MRSA and 55 being MSSA. The other 88 cases were infected with CoNS, of which 13 were MR-CoNS and 75 were MS-CoNS. Regarding surgical procedures, DAIR, one-stage revision, and two-stage revision were performed in 55 cases, 42 cases, and 51 cases, respectively. Among the patients in the non-Rifampicin treatment group, 7 were treated with cephalosporins, 109 with fluoroquinolones, and 18 with vancomycin based on the results of bacterial culture and drug susceptibility tests. The remaining 14 patients received other types of antibiotics.

**Table 1:** Demographic characteristics of all patients.

Variables	Rifampicin treatment group (*n* =79)	Non-rifampicin treatment group (*n* = 148)	p-value
Age (years)	65.44 ± 9.82	65.12 ± 10.43	0.822^a^
Sex (male/female), n	41/38	66/82	0.294^b^
Median BMI, kg/m^2^ (IQR)	25.60 (24.30, 26.90)	26.50 (24.53, 27.10)	0.055^e^
Comorbidities			
Diabetes mellitus, n	17	35	0.716^b^
Autoimmune disease, n	2	8	0.506^c^
Active cancer, n	2	2	0.616^d^
Active smoking, n	6	17	0.355^b^
Median CCI (IQR)	4.00 (4.00, 4.00)	4.00 (3.00, 5.00)	0.350^e^
Median ASA score (IQR)	2.00 (2.00, 2.00)	2.00 (2.00, 2.00)	0.456^e^
Joint (hip/knee), n	42/37	75/73	0.721^b^
Laboratory examination			
Median WBC, 10^9^/L (IQR)	7.98 (5.53, 9.77)	7.21 (5.58, 8.98)	0.187^e^
Median CRP, mg/L (IQR)	25.30 (9.90, 49.45)	25.00 (12.01, 59.61)	0.367^e^
Median ESR, mm/h (IQR)	48.00 (34.00, 70.00)	54.00 (41.00, 75.00)	0.210^e^
Median D-dimer, mg/L (IQR)	2.85 (1.83, 3.45)	2.38 (1.75, 3.25)	0.324^e^
Median IL-6, pg/mL (IQR)	25.32 (14.25, 39.48)	23.84 (15.54, 39.47)	0.610^e^
Median SF WBC,/mL (IQR)	10587.00 (5448.00, 18254.00)	10256.00 (6374.75, 17049.25)	0.978^e^
Median SF PMN, % (IQR)	86.50 (75.00, 92.10)	86.30 (75.05, 91.60)	0.900^e^
Microorganism			
*Staphylococcus aureus*, n	30	60	0.707^b^
MSSA, n	27	55	0.656^b^
MRSA, n	3	5	1.000^c^
CoNS, n	49	88	0.707^b^
MS-CoNS, n	42	75	0.721^b^
MR-CoNS, n	7	13	0.984^b^
Type of operation			
DAIR, n	33	55	0.497^b^
One-stage revision, n	21	42	0.773^b^
Two-stage revision, n	25	51	0.669^b^
Basic Antibiotic therapy			
Cephalosporin, n	3	7	1.000^c^
Fluoroquinolone, n	60	109	0.705^b^
Vancomycin, n	10	18	0.994^b^
Other antibiotics, n	6	14	0.637^b^
Drug-related adverse events, n	25	13	<0.001^b^
Drug-related adverse event leading to rifampin, n	8	0	<0.001^c^
Remission, n	62	100	0.083^b^
Median follow-up time, mths (IQR)	15.00 (14.00, 17.00)	15.00 (14.00, 17.00)	0.480^e^

a Independent-samples t-test.

b Pearson’s Chi-squared test.

c Chi-square test for continuity correction.

d Fisher’s exact test.

e Mann-Whitney U test.

BMI, body mass index; CCI, charlson comorbidity index; ASA, American Society of Anesthesiologists; WBC, white blood cell count; CRP, C-reactive protein; ESR, erythrocyte sedimentation rate; IL-6, interleukin-6; SF, synovial fluid; PMN, polymorphonuclear neutrophil; MSSA, methicillin-susceptible *Staphylococcus aureus*; MRSA, methicillin-resistant *Staphylococcus aureus*; CoNS, coagulase negative staphylococci; MS-CoNS, methicillin-susceptible coagulase negative staphylococci; MR-CoNS, methicillin-resistant coagulase negative staphylococci.

### 3.2 Bacterial distribution and the role of mNGS

**Figure 2:**
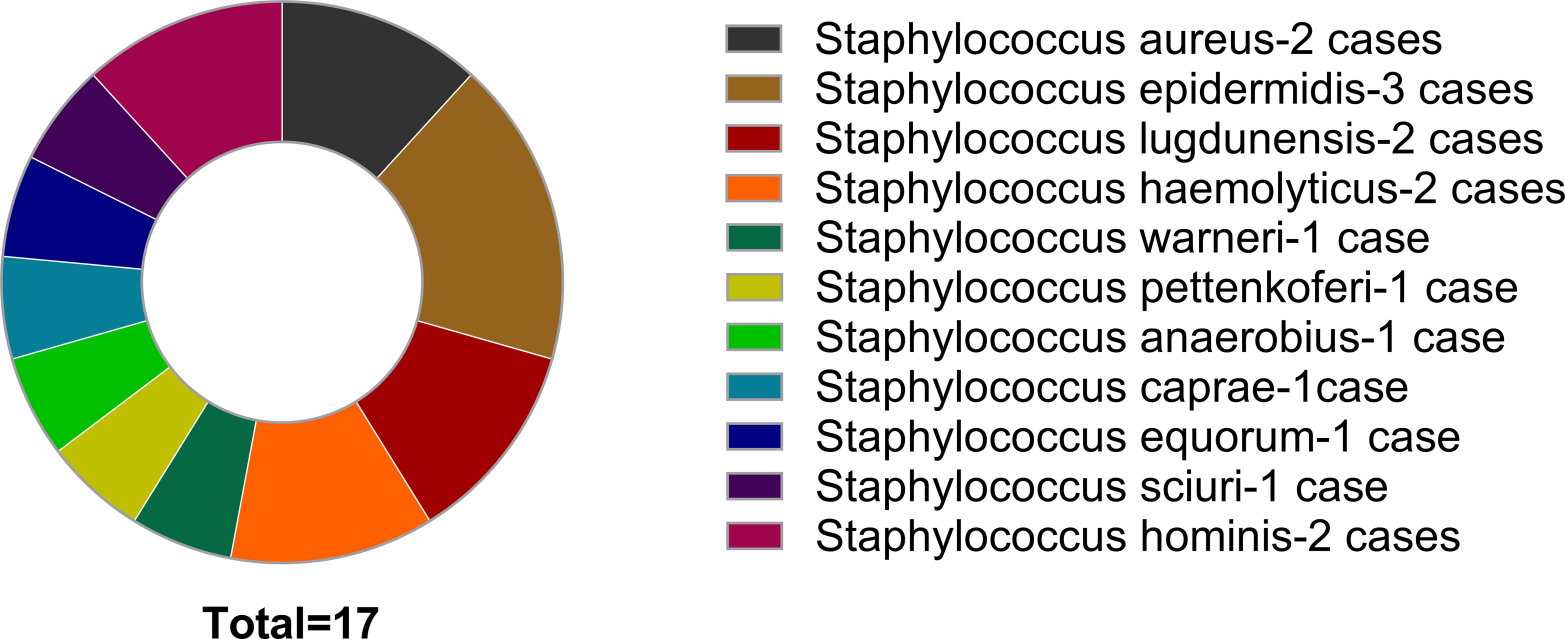
Bacteria cultured under the guidance of mNGS findings.

Among 227 patients, the majority of cases involved infections with *Staphylococcus aureus* and *Staphylococcus epidermidis*, amounting to 90 (36.65%) and 65 (28.63%) Cases Respectively. 17 patients initially yielded negative culture results; subsequent cultivation identified pathogens based on mNGS findings (**Figure 2**).

### 3.3 Efficacy of rifampin in treating staphylococcal PJI

There were no statistically significant differences between the rifampin treatment group and the non-rifampin treatment group in terms of demographic characteristics, infection sites, bacterial distribution, and surgical methods. The number of patients who achieved remission in the two groups were 62 (79.75%) and 100 (73.65%), respectively. There was no statistically significant difference in the remission rate between the two groups (p = 0.083, pearson’s chi-squared test) (**Table 1**).

**Figure 3:**
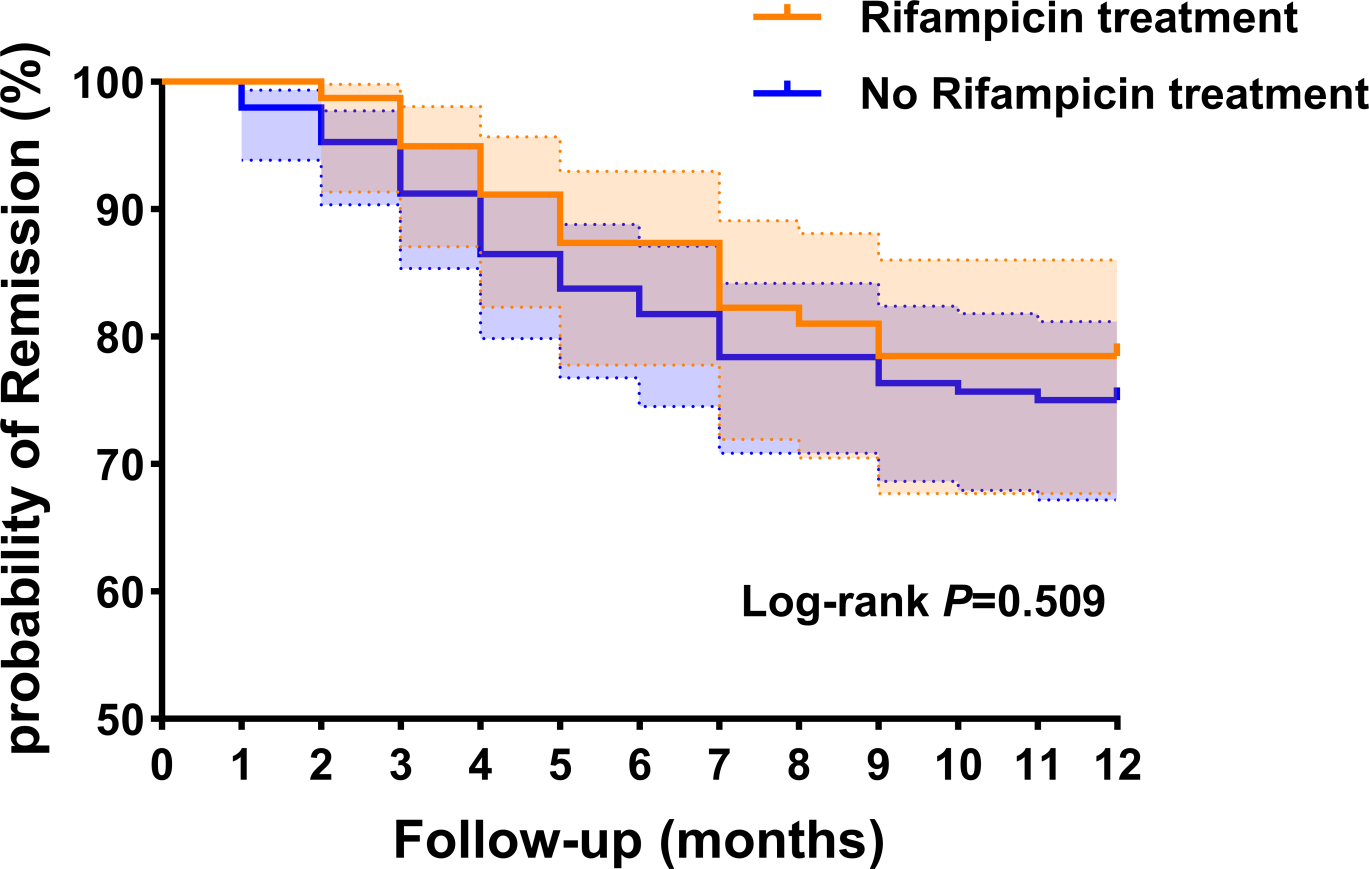
Kaplan-Meier survival curve analysis results of rifampicin treatment group and no rifampicin treatment group. This dotted line represents the 95% confidence interval.

We generated Kaplan-Meier survival curve analysis results of rifampicin treatment group and no rifampicin treatment group. The log-rank test was utilized to assess the differences in failure times between these two groups, revealing no statistically significant differences (p = 0.509, log-rank test) (**Figure 3**).

23 patients who received rifampin treatment but did not complete the full 12-week course were excluded from the rifampin treatment group, with seven of these cases resulting in treatment failure (**Figure 1**). The exclusion of patients with less than 12 weeks of rifampin therapy could potentially introduce bias, leading to an artificially higher survival rate in the rifampin group. To address this concern, we conducted a supplementary analysis comparing the response rates between the non-rifampin treatment group and a combined rifampin treatment group that included patients with less than 12 weeks of therapy. The revised analysis yielded results consistent with our preliminary findings (73.63% vs. 76.47%, p = 0.127).

To delve deeper into whether the administration of rifampicin influenced treatment outcomes across various surgical methods and sites, a subgroup analysis was performed. In the context of DAIR procedures, the overall remission rates for patients treated with rifampicin and those without were 75.76% and 61.82%, respectively. When analyzing by joint type, in the hip, these rates were 76.19% and 64.00%, and in the knee, they were 75.00% and 60.00%. Despite the lower remission rates in the non-rifampicin group compared to the rifampicin group, statistical significance was not achieved. In revision surgeries, the overall remission rates for the rifampicin and non-rifampicin groups were 80.43% and 81.72%, respectively. For the hip, these rates were 76.19% and 82.00%, and for the knee, they were 84.00% and 81.40%. In one-stage revisions, the rates were identical at 80.95% for both groups, and in the knee, they were 80.00% and 82.35%, again with no statistically significant differences (**Table 2**).

**Table 2:** Subgroup analysis of outcomes in rifampicin treatment and non-rifampicin treatment groups.

Subgroups	Rifampicin treatment group (remission/failure)	Non-rifampicin treatment group (remission/failure)	p-value
DAIR, n	25/8	34/21	0.178^a^
Joint			
Hip, n	16/5	16/9	0.371^a^
Knee, n	9/3	18/12	0.573^b^
Revision, n	37/9	76/17	0.855^a^
Joint			
Hip, n	16/5	41/9	0.814^b^
Knee, n	21/4	35/8	1.000^b^
Type of revision			
One-stage revision, n	17/4	34/8	1.000^b^
Two-stage revision, n	20/5	42/9	1.000^b^

a Pearson’s Chi-squared test.

b Continuity correction Chi-squared test.

### 3.4 Rifampin increases the incidence of drug-related adverse events

A total of 38 patients encountered drug-related adverse events. The incidence was markedly higher in the group that received rifampicin compared to those who did not (31.65% vs 8.78%, p < 0.001, pearson’s chi-squared test). Notably, rifampicin was identified as the direct cause in 8 of these cases (**Table 1**).

### 3.5 Analysis of factors associated with treatment failure in PJI

**Figure 4:**
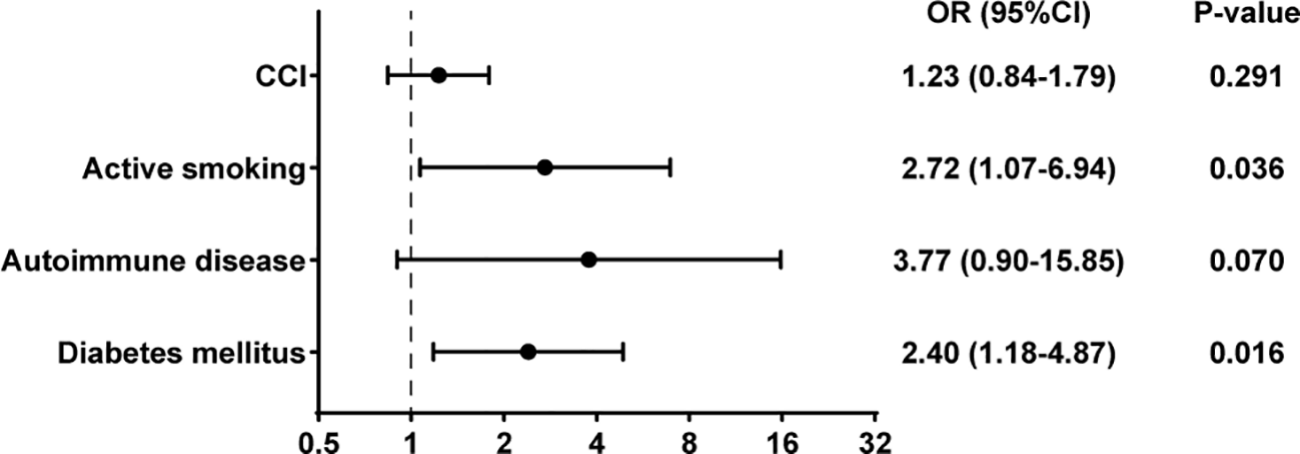
Multivariate binary logistic regression analysis of risk factors for treatment failure in PJI.

In the univariate binary logistic regression analysis, several factors were identified as influencers of treatment failure outcomes, including diabetes (Odds Ratio [OR], 2.59; 95% Confidence Interval [CI], 1.32-5.08; p=0.006), autoimmune disease (OR, 4.32; 95% CI, 1.12-16.68; p=0.034), active smoking (OR, 3.15; 95% CI, 1.32-7.52; p=0.010), and the CCI (OR, 1.48; 95% CI, 1.05-2.10; p=0.027) (**Table 3**). Upon incorporating these risk factors into a multivariate binary logistic regression analysis model, diabetes (OR, 2.40; 95% CI, 1.18-4.87; p=0.016) and active smoking (OR, 2.72; 95% CI, 1.07-6.94; p=0.036) emerged as independent and significant risk factors for treatment failure. Conversely, rifampicin was not found to be an independent risk factor impacting outcomes (**Figure 4**).

**Table 3:** Univariate binary logistic regression analysis of risk factors for clinical failure.

Variables	OR (95%CI)	p-value*
Age	0.99 (0.96-1.02)	0.430
Sex	1.43 (0.77-2.61)	0.269
BMI	0.87 (0.72-1.04)	0.130
Comorbidities		
Diabetes mellitus	2.59 (1.32-5.08)	0.006
Autoimmune disease	4.31 (1.12-16.68)	0.034
Active cancer	1.07 (0.11-10.50)	0.954
Active smoking	3.15 (1.32-7.52)	0.010
CCI	1.48 (1.05-2.10)	0.027
ASA score	1.37 (0.54-3.49)	0.514
Joint		
Hip	1.02 (0.55-1.87)	0.958
Knee	0.98 (0.53-1.81)	0.958
Laboratory examination		
WBC	1.04 (0.98-1.10)	0.182
CRP	1.01 (1.00-1.01)	0.126
ESR	1.00 (0.99-1.02)	0.478
D-dimer	0.97 (0.80-1.16)	0.705
IL-6	1.00 (0.99-1.01)	0.673
SF WBC	1.00	0.544
SF PMN	1.00 (0.98-1.03)	0.806
Microorganism		
* Staphylococcus aureus*	1.06 (0.57-1.98)	0.851
MSSA	0.95 (0.50-1.80)	0.869
MRSA	1.98 (0.46-8.56)	0.362
CoNS	0.94 (0.51-1.76)	0.851
MS-CoNS	0.76 (0.41-1.40)	0.378
MR-CoNS	1.83 (0.69-4.86)	0.223
Basic Antibiotic therapy		
Cephalosporin	0.79 (0.16-3.85)	0.774
Fluoroquinolone	0.54 (0.28-1.04)	0.065
Vancomycin	1.96 (0.84-4.54)	0.118
Other antibiotics	1.83 (0.69-4.86)	0.223
Rifampin treatment	0.82 (0.43-1.58)	0.558
Follow-up time	1.08 (0.96-1.21)	0.227

*Univariate binary logistic regression analysis.

OR, Odds Ratio; CI, Confidence Interval; BMI, body mass index; CCI, Charlson comorbidity index; ASA, American Society of Anesthesiologists; WBC, white blood cell count; CRP, C-reactive protein; ESR, erythrocyte sedimentation rate; IL-6, interleukin-6; SF, synovial fluid; PMN, polymorphonuclear neutrophil; MSSA, methicillin-susceptible *Staphylococcus aureus*; MRSA, methicillin-resistant *Staphylococcus aureus*; CoNS, Coagulase negative staphylococci; MS-CoNS, methicillin-susceptible coagulase negative staphylococci; MR-CoNS, methicillin-resistant coagulase negative staphylococci.

## 4 Discussion

Due to limited evidence supporting the use of rifampin in combination therapy for PJI caused by staphylococci, this was a retrospective observational study involving 227 patients with staphylococcal PJI. The aim of this study was to compare the impact of adding rifampin to sensitive antimicrobial therapy on the treatment outcomes of staphylococcal PJI. We found that the use of rifampin in addition to sensitive antibiotic therapy did not significantly differ in terms of the remission rate compared to those without rifampin. Additionally, there was no significant difference observed in the failure time between the two groups.

In our study, factors associated with failure in staphylococcal PJI included diabetes, autoimmune diseases, active smoking, and the CCI. However, in multivariate analysis, only diabetes and active smoking showed a significant positive correlation with the outcome of failure. This aligns with findings from other studies, which have also identified active smoking and diabetes as significant factor (Zimmerli et al., 1998; Lesens et al., 2018). Some studies have reported that the combination of rifampin and fluoroquinolones is a predictive factor for outcomes in PJI caused by *Staphylococcus aureus* (Senneville et al., 2011). In our study, however, the use of rifampin was not an independent predictor of outcome.

For acute PJI, the DAIR procedure is commonly used. Many researchers believe that rifampin plays a crucial role in DAIR, as it effectively inhibits the growth of residual bacteria and prevents the reformation of biofilms on the implant surface. Numerous studies have found that using a rifampin-containing regimen in conjunction with DAIR can significantly improve the rate of infection control (Tai et al., 2022; Cushing and Drekonja, 2023; Scheper et al., 2021; Yusuf et al., 2024). However, the reported failure rates for this strategy vary widely, ranging from 10% to 70%, due to differences in patient comorbidities, joint types, surgical techniques, and antimicrobial strategies (Kunutsor et al., 2018; Gerritsen et al., 2021). In our study, the success rate of DAIR for acute staphylococcal PJI was 75.76% in the rifampin treatment group and 61.82% in the non-rifampin treatment group. These results are similar to those reported by Karlsen et al., who found a 2-year success rate of 74% in the rifampin combination group and 72% in the monotherapy group (Karlsen et al., 2020). Although the remission rate was higher in the rifampin group, there was no statistically significant difference. The implant can be retained because the infection duration is short, and bacteria have not yet fully penetrated the surrounding tissue. Although biofilms can begin to form within one day, their impact on surrounding tissues may be limited in the early stages of infection. DAIR is an emergency procedure that involves thorough debridement surgery to remove infected tissue and reduce bacterial load, thereby disrupting the biofilm and preventing its further development. In our cases, all DAIR surgeries were performed by an experienced senior surgeon using standardized procedures. During surgery, all infected and necrotic tissue were removed, t liners or femoral heads were implanted.

For chronic infections or as salvage therapy, most cases undergo either one-stage or two-stage revision surgery (Anemüller et al., 2019). There is ongoing debate about whether rifampin is necessary in revision surgeries where the implant is removed. A multicenter observational study by Kramer et al. found no significant difference in failure rates between patients who received rifampin with antibiotics after revision surgery and those who did not (22.5% vs. 31.4%). They suggest that once the implant is completely removed, rifampin may not be necessary (Kramer et al., 2023). Studies by Junyent J and Rajgopal A also support this view (Gómez-Junyent et al., 2021; Rajgopal et al., 2018). After thorough debridement, the biofilm is largely removed, the biofilm is largely removed, making a clinical cure of infection more likely. Some studies indicate that staphylococci can not only form biofilms but also invade osteoblasts and bone cells, leading to intracellular infections, particularly with *Staphylococcus aureus*. These bacteria can escape from endosomes before lysosome fusion or survive in phagolysosomes, multiplying in the cytoplasm or vacuoles (Schröder et al., 2006; Flannagan et al., 2016). Due to intracellular virulence factors such as phenol-soluble modulins (PSMs) or protein A, *Staphylococcus aureus* infections can also induce osteoblast death (Davido et al., 2016). In addition to contributing to bone destruction, cell lysis allows bacteria to release into the extracellular medium, infecting new host cells and perpetuating the infection (Jubrail et al., 2016). Furthermore, *Staphylococcus aureus* has been observed to undergo phenotypic shifts to small colony variant (SCV) and persisters within cells, which are associated with better intracellular persistence due to their reduced virulence (Schröder et al., 2006). These factors are related to treatment failures in chronic staphylococcal PJI. Since rifampin can not only inhibit biofilms but also accumulate intracellularly (Valour et al., 2015), many researchers believe that its use in combination is still important. In our study, there was no significant difference in success rates between treatments with and without rifampin in one-stage revisions, two-stage revisions, and in both hip and knee joint revisions. This may have been due to the thorough debridement performed during surgery, which effectively removed biofilms, infected bone cells, and osteoblasts. Additionally, our excellent culture techniques had improved the detection rates of pathogens, allowing for the selection of sensitive antibiotics to prevent the formation of new biofilms on the surfaces of new implants or spacers.

Microbial culture plays a crucial role in the diagnosis and treatment of PJI. We follow a standardized procedure for culture, extending the culture duration when necessary. For challenging cases, we combine mNGS and PCR testing. By employing precise pathogen diagnosis strategies, we largely rule out mixed infections and infections from bacteria in special states, such as SCV and viable but non-culturable states, allowing for targeted antibiotic therapy. It should be emphasized that mNGS is not employed as a diagnostic standard for PJI. Rather, it serves as an adjunctive tool to augment the diagnostic rate, especially in cases where conventional cultures are negative. Moreover, the possibility of false-positive results with mNGS requires careful consideration. Combined with standardized surgical procedures, the bacterial load is kept low, thus achieving good therapeutic effects. In such cases, the clinical significance of combining rifampin may not be substantial. The use of rifampin can lead to resistance and potentially increase the incidence of rifampin-related complications.

The implementation of an antibiotic holiday in the treatment of PJI remains a contentious issue within the orthopedic community, with significant variability in the antibiotic holidays employed by surgeons. The interval between stages ranges from 2 weeks to several months (Lichstein et al., 2014). Lombardi et al. observed an 89% success rate for two-stage revision hip arthroplasty with an antibiotic holiday between 6 and 12 weeks (Lombardi et al., 2014). Klouche et al., using a 6-week antibiotic holiday, reported a 97.8% infection eradication rate for two-stage revision hip arthroplasty (Klouche et al., 2012). In contrast, Bejon et al. investigated two-stage revisions with at least a 2-week antibiotic holiday before reimplantation over a 4-year period and concluded that an antibiotic holiday was unnecessary (Bejon et al., 2010). The International Consensus Meeting does not recommend an antibiotic holiday before reimplantation, citing a lack of evidence to support this practice (Restrepo et al., 2014). While cessation of antibiotics may be necessary to accurately assess infection status and reduce the risk of developing antibiotic-resistant strains, it also poses the potential risk of infection recurrence or exacerbation. Studies have shown that an antibiotic holiday does not affect the success of PJI treatment in patients undergoing reimplantation. However, many patients experience failure during the antibiotic holiday (Tan et al., 2018). In our study, employing a 4-week antibiotic holiday, two patients experienced failure during this period. Therefore, the use of an antibiotic holiday should be tailored based on the characteristics of the infecting organism, the adequacy of debridement, and the individual patient’s condition.

This study has several limitations. It was a non-randomized and retrospective study with a small overall sample size, particularly in the rifampicin treatment group. Given that patients who did not complete the 12-week rifampin treatment were excluded, the results may be subject to bias. Therefore, our findings may not be fully generalizable to all patient populations, especially among those with treatment discontinuation. Additionally, the surgical sites included in our cases were limited to the hip and knee, making it difficult to determine whether rifampin offers potential benefits in other anatomical locations. To further explore the efficacy and complications of rifampin in treating staphylococcal PJI, we initiated a nationwide multicenter, prospective randomized controlled clinical trial in October 2024, focusing on the effectiveness and complications of rifampin combined with antibiotics for staphylococcal PJI.

## 5 Conclusion

In summary, our study results indicated that the addition of rifampin to the standard antibiotic treatment for staphylococcal PJI did not affect the final remission rate or the time to failure outcome. This held true regardless of whether the treatment involved DAIR or one-stage or two-stage revision, and applied to both hip and knee joints. The use of rifampin significantly increased the risk of drug side effects, and its adjunctive use did not provide the expected benefits. Standard surgical procedures, accurate pathogen diagnosis, and treatment are particularly crucial in the management of PJI.

## Data Availability

The raw data supporting the conclusions of this article will be made available by the authors, without undue reservation.

## References

[B1] Aggarwal V. Bakhshi H. Ecker N. Parvizi J. Gehrke T. Kendoff D. (2014). Organism profile in periprosthetic joint infection: pathogens differ at two arthroplasty infection referral centers in europe and in the United States. J. Knee Surg. 27, 399–4065. doi: 10.1055/s-0033-1364102, PMID: 24414388

[B2] Anemüller R. Belden K. Brause B. Citak M. Pozo J. L.D. Frommelt L.. (2019). Hip and knee section, treatment, antimicrobials: proceedings of international consensus on orthopedic infections. J. Arthroplasty 34, S463–S475. doi: 10.1016/j.arth.2018.09.032, PMID: 30348582

[B3] Aydın O. Ergen P. Ozturan B. Ozkan K. Arslan F. Vahaboglu H. (2021). Rifampin-accompanied antibiotic regimens in the treatment of prosthetic joint infections: A frequentist and bayesian meta-analysis of current evidence. Eur. J. Clin. Microbiol. Infect. Dis. 40, 665–715. doi: 10.1007/s10096-020-04083-4, PMID: 33125602

[B4] Baciewicz A. M. Chrisman C. R. Finch C. K. Self. T. H. (2013). Update on rifampin, rifabutin, and rifapentine drug interactions. Curr. Med. Res. Opin. 29, 1–125. doi: 10.1185/03007995.2012.747952, PMID: 23136913

[B5] Bejon P. Berendt A. Atkins B. L. Green N. Parry H. Masters S.. (2010). Two-stage revision for prosthetic joint infection: predictors of outcome and the role of reimplantation microbiology. J. Antimicrob. Chemother. 65, 569–575. doi: 10.1093/jac/dkp469, PMID: 20053693 PMC2818105

[B6] Beldman M. Löwik C. Soriano A. Albiach L. Zijlstra W. P. Knobben B. A.S.. (2021). If, when, and how to use rifampin in acute staphylococcal periprosthetic joint infections, a multicentre observational study. Clin. Infect. Dis. 73, 1634–1641. doi: 10.1093/cid/ciab426, PMID: 33970214 PMC8563307

[B7] Cobo J. Del Pozo J. L. (2011). Prosthetic joint infection: diagnosis and management. Expert Rev. Anti-Infective Ther. 9, 787–8025. doi: 10.1586/eri.11.95, PMID: 21905787

[B8] Cushing S. Drekonja D. (2023). Rifampin for prosthetic joint infections: lessons learned over 20 years at a VA medical center. Federal Practit. 40, 294–995. doi: 10.12788/fp.0406, PMID: 38562157 PMC10984691

[B9] Davido B. Saleh-Mghir A. Laurent F. Danel C. Couzon F. Gatin L.. (2016). Phenol-soluble modulins contribute to early sepsis dissemination not late local USA300-osteomyelitis severity in rabbits. PLoS One 11, e01571335. doi: 10.1371/journal.pone.0157133, PMID: 27275944 PMC4898696

[B10] Flannagan R. S. Heit B. Heinrichs D. E. (2016). Intracellular replication of staphylococcus aureus in mature phagolysosomes in macrophages precedes host cell death, and bacterial escape and dissemination. Cell. Microbiol. 18, 514–355. doi: 10.1111/cmi.12527, PMID: 26408990

[B11] Gerritsen M. Khawar A. Scheper H. Wal R. v. d. Schoones J. de Boer M.. (2021). Modular component exchange and outcome of DAIR for hip and knee periprosthetic joint infection: A systematic review and meta-regression analysis. Bone Joint Open 2, 806–125. doi: 10.1302/2633-1462.210.BJO-2021-0090.R1, PMID: 34592839 PMC8558449

[B12] Gómez-Junyent J. Lora-Tamayo J. Baraia-Etxaburu J. Sánchez-Somolinos M. Iribarren J. A. Rodriguez-Pardo D.. (2021). Implant removal in the management of prosthetic joint infection by staphylococcus aureus: outcome and predictors of failure in a large retrospective multicenter study. Antibiot. (Basel Switzerland) 10, 118. doi: 10.3390/antibiotics10020118, PMID: 33530523 PMC7911003

[B13] Helou O. C. Berbari E. F. Lahr B. D. Eckel-Passow J. E. Razonable R. R. Sia I. G.. (2010). Efficacy and safety of rifampin containing regimen for staphylococcal prosthetic joint infections treated with debridement and retention. Eur. J. Clin. Microbiol. Infect. Dis. 29, 961–967. doi: 10.1007/s10096-010-0952-9, PMID: 20505968

[B14] Hu H. Ding H. Lyu J. Chen Y. Huang C. Zhang C.. (2024). Detection of rare microorganisms in bone and joint infections by metagenomic next-generation sequencing. Bone Joint Res. 13, 401–105. doi: 10.1302/2046-3758.138.BJR-2023-0420.R1, PMID: 39142657 PMC11324352

[B15] Jubrail J. Morris P. Bewley M. A. Stoneham S. Johnston S. A. Foster S. J.. (2016). Inability to sustain intraphagolysosomal killing of staphylococcus aureus predisposes to bacterial persistence in macrophages. Cell. Microbiol. 18, 80–965. doi: 10.1111/cmi.12485, PMID: 26248337 PMC4778410

[B16] Karlsen ØE. Borgen Pål. Bragnes B. Figved W. Grøgaard B. Rydinge J.. (2020). Rifampin combination therapy in staphylococcal prosthetic joint infections: A randomized controlled trial. J. Orthopaedic Surg. Res. 15, 365. doi: 10.1186/s13018-020-01877-2, PMID: 32859235 PMC7455995

[B17] Klouche S. Leonard P. Zeller V. Lhotellier L. Graff W. Leclerc P.. (2012). Infected total hip arthroplasty revision: One- or two-stage procedure? Orthop. Traumatol. Surg. Res. 98 (2), 144–50. doi: 10.1016/j.otsr.2011.08.018, PMID: 22364829

[B18] Kramer T. S. Soriano A. Tedeschi S. Chen A. F. Tattevin P. Senneville E.. (2023). Should we use rifampicin in periprosthetic joint infections caused by staphylococci when the implant has been exchanged? A multicenter observational cohort study. Open Forum Infect. Dis. 10, ofad491. doi: 10.1093/ofid/ofad491, PMID: 37901121 PMC10604993

[B19] Kunutsor S. K. Beswick A. D. Whitehouse M. R. Wylde V. Blom. A. W. (2018). Debridement, antibiotics and implant retention for periprosthetic joint infections: A systematic review and meta-analysis of treatment outcomes. J. Infection 77, 479–885. doi: 10.1016/j.jinf.2018.08.017, PMID: 30205122

[B20] Lesens O. Ferry T. Forestier E. Botelho-Nevers E. Pavese P. Piet E.. (2018). Should we expand the indications for the DAIR (Debridement, antibiotic therapy, and implant retention) procedure for staphylococcus aureus prosthetic joint infections? A multicenter retrospective study. Eur. J. Clin. Microbiol. Infect. Dis. 37, 1949–1956. doi: 10.1007/s10096-018-3330-7, PMID: 30083889

[B21] Lichstein P. Gehrke T. Lombardi A. Romano C. Stockley I. Babis G.. (2014). One-stage vs two-stage exchange. J. Arthroplasty 29, 108–111. doi: 10.1016/j.arth.2013.09.048, PMID: 24360339

[B22] Lombardi A. V. Berend K. R. Adams J. B. (2014). Partial two-stage exchange of the infected total hip replacement using disposable spacer moulds. Bone Joint J. 96, 66–69. doi: 10.1302/0301-620X.96B11.34360, PMID: 25381411

[B23] Lora-Tamayo J. Murillo O. Iribarren J. A. Soriano A. Sánchez-Somolinos M. Baraia-Etxaburu J. M.. (2013). A large multicenter study of methicillin-susceptible and methicillin-resistant staphylococcus aureus prosthetic joint infections managed with implant retention. Clin. Infect. Dis. 56, 182–194. doi: 10.1093/cid/cis746, PMID: 22942204

[B24] Mohamed W. Sommer U. Sethi S. Domann E. Thormann U. Schütz I.. (2014). Intracellular proliferation of S. Aureus in osteoblasts and effects of rifampicin and gentamicin on S. Aureus intracellular proliferation and survival. Eur. Cells Mater. 28, 258–268. doi: 10.22203/ecm.v028a18, PMID: 25340805

[B25] Osmon D. R. Berbari E. F. Berendt A. R. Lew D. Zimmerli W. Steckelberg J. M.. (2013). Diagnosis and management of prosthetic joint infection: clinical practice guidelines by the infectious diseases society of America. Clin. Infect. Dis. 56, e1–255. doi: 10.1093/cid/cis803, PMID: 23223583

[B26] Parvizi J. Zmistowski B. Berbari E. F. Bauer T. W. Springer B. D. Valle C. J.D.. (2011). New definition for periprosthetic joint infection: from the workgroup of the musculoskeletal infection society. Clin. Orthopaedics Related Res. 469, 2992–2945. doi: 10.1007/s11999-011-2102-9, PMID: 21938532 PMC3183178

[B27] Rajgopal A. Panda I. Rao A. Dahiya V. Gupta H. (2018). Does prior failed debridement Compromise the outcome of subsequent two-stage revision done for periprosthetic joint infection following total knee arthroplasty? J. Arthroplasty 33, 2588–2945. doi: 10.1016/j.arth.2018.02.087, PMID: 29627258

[B28] Renz N. Trampuz A. (2015). Periprothetische Infektionen: aktueller Stand der Diagnostik und Therapie. Orthopädie Rheuma 18, 20–285. doi: 10.1007/s15002-015-0779-y

[B29] Restrepo C. Schmitt S. Backstein D. Alexander B. T. Babic M. Brause B. D.. (2014). Antibiotic treatment and timing of reimplantation. J. Orthopaedic Res. 32, S136–S140. doi: 10.1002/jor.22557, PMID: 24464887

[B30] Scheper H. Gerritsen L. M. Pijls B. G. Van Asten S. A. Visser L. G. De Boer M. G. J. (2021). Outcome of debridement, antibiotics, and implant retention for staphylococcal hip and knee prosthetic joint infections, focused on rifampicin use: A systematic review and meta-analysis. Open Forum Infect. Dis. 8, ofab298. doi: 10.1093/ofid/ofab298, PMID: 34258321 PMC8271145

[B31] Schröder A. Kland R. Peschel A. Eiff C. v. Aepfelbacher. M. (2006). Live cell imaging of phagosome maturation in staphylococcus aureus infected human endothelial cells: small colony variants are able to survive in lysosomes. Med. Microbiol. Immunol. 195, 185–945. doi: 10.1007/s00430-006-0015-0, PMID: 16596413

[B32] Senneville E. Joulie D. Legout L. Valette M. Dezèque H. Beltrand E.. (2011). Outcome and predictors of treatment failure in total hip/knee prosthetic joint infections due to staphylococcus aureus. Clin. Infect. Dis. 53, 334–340. doi: 10.1093/cid/cir402, PMID: 21810745 PMC3148259

[B33] Tai D. B. G. Berbari E. F. Suh G. A. Lahr B. D. Abdel M. P. Tande A. J. (2022). Truth in DAIR: duration of therapy and the use of quinolone/rifampin-based regimens after debridement and implant retention for periprosthetic joint infections. Open Forum Infect. Dis. 9, ofac363. doi: 10.1093/ofid/ofac363, PMID: 36072695 PMC9439576

[B34] Tan T. L. Kheir M. M. Rondon A. J. Parvizi J. George J. Higuera C. A.. (2018). Determining the role and duration of the ‘Antibiotic holiday’ Period in periprosthetic joint infection. J. Arthroplasty 33, 2976–2805. doi: 10.1016/j.arth.2018.04.019, PMID: 29866503

[B35] Tande A. J. Patel R. (2014). Prosthetic joint infection. Clin. Microbiol. Rev. 27, 302–455. doi: 10.1128/CMR.00111-13, PMID: 24696437 PMC3993098

[B36] Valour F. Trouillet-Assant S. Riffard N. Tasse J. Flammier S. Rasigade J.-P.. (2015). Antimicrobial activity against intraosteoblastic staphylococcus aureus. Antimicrob. Agents Chemother. 59, 2029–2365. doi: 10.1128/AAC.04359-14, PMID: 25605365 PMC4356812

[B37] Wehrli W. (1983). Rifampin: mechanisms of action and resistance. Rev. Infect. Dis. 5, S407–S411. doi: 10.1093/clinids/5.supplement_3.s407, PMID: 6356275

[B38] Widmer A. F. Frei R. Rajacic Z. Zimmerli W. (1990). Correlation between *in vivo* and *in vitro* efficacy of antimicrobial agents against foreign body infections. J. Infect. Dis. 162, 96–102. doi: 10.1093/infdis/162.1.96, PMID: 2355207

[B39] Wu B. Su J. Zhang Z. Zeng J. Fang X. Li W.. (2024). Prosthetic spacers in two-stage revision for knee periprosthetic joint infection achieve better function and similar infection control. Bone Joint Res. 13, 306–145. doi: 10.1302/2046-3758.136.BJR-2023-0251.R1, PMID: 38889904 PMC11188966

[B40] Xu Z. Huang C. Lin Y. Chen Y. Fang X. Huang Z.. (2022). Clinical outcomes of culture-negative and culture-positive periprosthetic joint infection: similar success rate, different incidence of complications. Orthopaedic Surg. 1, 1420–1275. doi: 10.1111/os.13333, PMID: 35678131 PMC9251293

[B41] Yoon H.-K. Cho S.-H. Lee D.-Y. Kang B.-H. Lee S.-H. Moon D.-G.. (2017). A review of the literature on culture-negative periprosthetic joint infection: epidemiology, diagnosis and treatment. Knee Surg. Related Res. 29, 155–645. doi: 10.5792/ksrr.16.034, PMID: 28854760 PMC5596406

[B42] Yusuf E. Bramer W. Anas. A. A. (2024). Clinical outcomes of rifampicin combination therapy in implant-associated infections due to staphylococci and streptococci: A systematic review and meta-analysis. Int. J. Antimicrob. Agents 63, 1070155. doi: 10.1016/j.ijantimicag.2023.107015, PMID: 37875179

[B43] Zimmerli W. Widmer A. F. Blatter M. Frei R. Ochsner P. E. (1998). Role of rifampin for treatment of orthopedic implant-related staphylococcal infections: A randomized controlled trial. Foreign-body infection (FBI) study group. JAMA 279, 1537–1541. doi: 10.1001/jama.279.19.1537, PMID: 9605897

